# Applied pharmacogenetics to predict response to treatment of first psychotic episode: study protocol

**DOI:** 10.3389/fpsyt.2024.1497565

**Published:** 2025-01-07

**Authors:** Sergi Mas, Laura Julià, Manuel J. Cuesta, Benedicto Crespo-Facorro, Javier Vázquez-Bourgon, Carlos Spuch, Ana Gonzalez-Pinto, Angela Ibañez, Judith Usall, Cristina Romero-López-Alberca, Ana Catalan, Anna Mané, Miquel Bernardo

**Affiliations:** ^1^ Department of Clinical Foundations, Pharmacology Unit, University of Barcelona, Barcelona, Spain; ^2^ Institut d’investigacions Biomèdiques August Pi i Sunyer (IDIBAPs), Barcelona, Spain; ^3^ Centro de Investigación Biomédica en Red en Salud Mental (CIBERSAM), Madrid, Spain; ^4^ Departamento de Psiquiatría, Hospital Universitario de Navarra, Pamplona, Spain; ^5^ Instituto de Investigación Sanitaria de Navarra (IdiSNA), Pamplona, Spain; ^6^ Unidad de Gestión Clínica de Salud Mental, Hospital Universitario Virgen del Rocío, Sevilla, Spain; ^7^ Instituto de Biomedicina de Sevilla (IBiS), Hospital Universitario VIrgen del Rocio/Centro Superior de Investigaciones Cinetíficas (HUVR/CSIC)/Universidad de Sevilla, Seville, Spain; ^8^ Department of Psychiatry, Universidad de Sevilla, Seville, Spain; ^9^ Departamento de Psiquaitria, Marqués de Valdecilla University Hospital – IDIVAL, Santander, Spain; ^10^ Departamento de Medicina y Psiquiatria, Universidad de Cantabria, Santander, Spain; ^11^ Translational Neuroscience Research Group, Galicia Sur Health Research Institute (IIS-Galicia Sur), Servizo Galego de Saúde/Universidad de Vigo (SERGAS-UVIGO), Vigo, Spain; ^12^ BIOARABA, Department Psychiatry, Hospital Universitario Alava, Universidad del País Vasco/Euskal Herriko Unibertsitatea Vitoria (UPV/EHU), Vitoria, Spain; ^13^ Department of Psychiatry, Hospital Universitario Ramón y Cajal, Universidad de Alcalá, Instituto Ramón y Cajal de Investigación Sanitaria (IRYCIS), Madrid, Spain; ^14^ Institut de Recerca Sant Joan de Déu, Esplugues de Llobregat, Barcelona, Spain; ^15^ Centre de Salut Mental, Parc Sanitari Sant Joan de Déu, Sant Boi de Llobregat, Barcelona, Spain; ^16^ Departament de Ciències Experimentals i de la Salut, Department of Psychology, University of Cadiz, Cádiz, Spain; ^17^ Psychiatry Department, Basurto University Hospital, Osakidetza, Basque Health Service, Bilbao, Spain; ^18^ Biobizkaia Health Research Institute, OSI Bilbao-Basurto, Bilbao, Spain; ^19^ Neuroscience Department, University of the Basque Country, Leioa, Spain; ^20^ Department of Psychosis Studies, Institute of Psychiatry, Psychology & Neuroscience, King’s College London, London, United Kingdom; ^21^ Institut de Salud Mental, Hospital del Mar, Barcelona, Spain; ^22^ Hospital del Mar Research Institute, Barcelona, Spain; ^23^ Universitat Pompeu Fabra (UPF), Barcelona, Spain; ^24^ Barcelona Clinic Schizophrenia Unit, Hospital Clínic de Barcelona, Barcelona, Spain; ^25^ Departament de Medicina, Institut de Neurociències (UBNeuro), Universitat de Barcelona (UB), Barcelona, Spain

**Keywords:** personalized medicine, antipsychotic, prediction, psychosis, Pharmacogenetics

## Abstract

The application of personalized medicine in patients with first-episode psychosis (FEP) requires tools for classifying patients according to their response to treatment, considering both treatment efficacy and toxicity. However, several limitations have hindered its translation into clinical practice. Here, we describe the rationale, aims and methodology of *Applied Pharmacogenetics to Predict Response to Treatment of First Psychotic Episode* (the FarmaPRED-PEP project), which aims to develop and validate predictive algorithms to classify FEP patients according to their response to antipsychotics, thereby allowing the most appropriate treatment strategy to be selected. These predictors will integrate, through machine learning techniques, pharmacogenetic (measured as polygenic risk scores) and epigenetic data together with clinical, sociodemographic, environmental, and neuroanatomical data. To do this, the FarmaPRED-PEP project will use data from two already recruited cohorts: the PEPS cohort from the “Genotype-Phenotype Interaction and Environment. Application to a Predictive Model in First Psychotic Episodes” study (the PEPs study from the Spanish abbreviation) (N=335) and the PAFIP cohort from “Clinical Program on Early Phases of Psychosis” (PAFIP from the Spanish abbreviation) (N = 350). These cohorts will be used to create the predictor, which will then be validated in a new cohort, the FarmaPRED cohort (N = 300). The FarmaPRED-PEP project has been designed to overcome several of the limitations identified in pharmacogenetic studies in psychiatry: (1) the sample size; (2) the phenotype heterogeneity and its definition; (3) the complexity of the phenotype and (4) the gender perspective. The global reach of the FarmaPRED-PEP project is to facilitate the effective deployment of precision medicine in national health systems.

## Introduction

Schizophrenia (SZ) is the most paradigmatic psychotic disorder. The course of the illness is often chronic and highly variable, causing a significant loss of quality of life for the patient and their family members (1). It also has a high cost for society, accounting for 10% of the global burden of mental disorders in Europe (2). There is great variability in the efficacy of antipsychotic drugs (APs) in the treatment of SZ as well as in the susceptibility of patients to the side effects of these drugs. On average, between 20% and 30% of patients do not respond appropriately to AP treatment and less than 40% achieve symptom remission (3), with a 70% treatment discontinuation rate. This leads to relapses that entail new admissions, thereby worsening the prognosis, negatively affecting the patient’s quality of life, and reducing life expectancy (4). Furthermore, approximately 30% of patients develop treatment-resistant SZ (TRS). It is essential to elucidate the underlying pathophysiology of TRS to identify biomarkers for its early detection and treatment. As an example, plasma levels of metabolites involved in the Kynurenine pathway, at the crossroad between neuroinflammation and glutamatergic neurotransmission, has been proposed as biomarker of TRS (5). However, the rates of long-term recovery for first-episode psychosis (FEP) are more favorable since about 51% of patients may achieve either symptomatic, functional, and personal recovery (6). Selecting the best compound for each patient is a challenging procedure (7), and its selection, unfortunately, largely relies on clinical experience and a trial-and-error strategy that exposes the patients to a higher risk of adverse reactions, prolongs the recovery time, and worsens the long-term response (8).

In this context, the search for biomarkers to select the most suitable AP for each patient in the early stages is a priority area in psychiatry (9). Pharmacogenetics (PGx) has become one of the main tools in this search for biomarkers (3). However, PGx results are characterized by a lack of replicability, thus limiting their translation into clinical practice. These studies have relied on the knowledge of pharmacokinetic and/or pharmacodynamic processes. The pharmacokinetic studies have shown the most promising results for clinical implementation as the genetic variability in metabolism-related genes explains a significant percentage of the variability in the plasma levels of some APs. Genotyping these genes can be very useful in improving APs efficacy and tolerability (10). In this regard, international guideline groups, such as the Dutch Pharmacogenetics Working Group (DPWG), have published pharmacogenetic guidelines for gene-drug interactions involving CYP2D6, CYP3A4, and CYP1A2 with antipsychotics (11), while the Clinical Pharmacogenetics Implementation Consortium (CPIC) is developing similar guidelines (https://cpicpgx.org/prioritization-of-cpic-guidelines/). Additionally, regulatory agencies like the Food and Drug Administration (FDA) have identified 41 pharmacogenomic biomarkers relevant to psychiatry in drug labeling (https://www.fda.gov/drugs/science-and-research-drugs/table-pharmacogenomic-biomarkers-drug-labeling). By contrast, the pharmacodynamic studies have been rarely replicated in independent populations, often explaining a small percentage of the observed variability that limits their predictive capacity (12).

The candidate gene approach is further limited by the lack of understanding of the mechanism of action of the APs. The pharmacological response to APs can be defined as a complex phenotype with polygenic inheritance involving multiple loci with small effects. Polygenic risk scores (PRS) are constructed by summing the multiple risk alleles associated with a phenotype and are weighted by the magnitude of their estimated effect in genome-wide association studies (GWAS) using large cohorts with sufficient statistical power (13). In psychiatry, these PRS have been calculated to estimate the genetic risk for various conditions, such as SZ, major depression, and bipolar disorder (14). Several studies have demonstrated that the PRS calculated for psychopathologies can be useful in pharmacogenomic studies (14–17) as predictors of the response to APs (18–22). Additionally, the PRS calculated for phenotypes related to the pharmacological response to APs, such as cognitive performance or metabolic alterations, can also be associated with the efficacy or toxicity of AP treatments (14, 23–25). The clinical application of PRS and their inclusion in healthcare systems are some of the most promising aspects in implementing PGx in clinical practice (13). However, PRS have limited precision in their predictive capacity, as there are many other sociodemographic, environmental, clinical, and pharmacological factors involved in this complex phenotype. Despite these limitations, there is evidence for the clinical applicability of PRS, mainly for patient identification and stratification (13, 17). In these examples, PRS have been incorporated into risk algorithms and are already used in clinical practice in some cases, increasing their predictive capacity. The integration of PRS into algorithms that include other risk factors (i.e., sociodemographic, environmental, clinical, and pharmacological) represents the future of personalized medicine.

### Applied pharmacogenetics to predict response to treatment of first psychotic episode

PGx studies have been hindered by limitations such as difficulties in recruiting large cohorts, as they require detailed information and longitudinal follow-up to assess the response to pharmacological treatment. Additionally, PGx studies in SZ are constrained by disease-specific limitations (14) such as diagnostic heterogeneity (multiple and variable symptoms accompanied by neuropsychological and functional impairments) (26), multiple comorbidities, and pharmacological heterogeneity (multiple APs with different properties, variable doses, AP combinations, and concomitant medications) (27, 28). Furthermore, there is heterogeneity in the definition of response phenotypes that is usually defined as a percentage of improvement in overall symptomatology or a threshold value measured in cross-sectional studies, without the consideration of the different symptoms and dimensions of psychiatric disorders, their longitudinal evolution, or years of treatment. Accordingly, several authors proposed adding functioning and personal recovery measures to the list of outcome indicators for FEP, expanding it beyond symptomatologic remission (29).

The “Applied Pharmacogenetics to Predict Response to Treatment of First Psychotic Episode” (FarmaPRED-PEP from the Spanish abbreviation) project is a multicenter study designed to allow the development and validation of a predictive algorithm for the application of personalized medicine in patients with first-episode psychosis (FEP). The purpose of this predictor will be to classify FEP patients according to their response phenotype to APs, thereby allowing the most appropriate treatment strategy to be selected. This predictor will integrate, through machine learning techniques, pharmacogenetic (measured as polygenic risk scores) and epigenetic data with clinical, sociodemographic, environmental, and neuroanatomical data. To do this, the FarmaPRED-PEP project will use the data from two already recruited cohorts of patients with FEP and a longitudinal follow-up: the PEPS cohort from “Genotype-Phenotype Interaction and Environment. Application to a Predictive Model in First Psychotic Episodes” (the PEPs study from the Spanish abbreviation; PI08/0208) (N = 335) (30) and the PAFIP cohort from “Clinical Program on Early Phases of Psychosis” (PAFIP from the Spanish abbreviation)” (N = 350) (31). These cohorts will allow response phenotypes to be defined using longitudinal data, taking into account not only the symptomatological dimensions of the pathology, but also the neurocognitive dimensions and adverse effects. These cohorts will be used to create as well as internally validate the predictor. This predictor will be externally validated in a new prospective cohort of patients with FEP and a longitudinal follow-up, the FarmaPRED cohort (N = 300).

## Study design

### Study design

**Figure 1:**
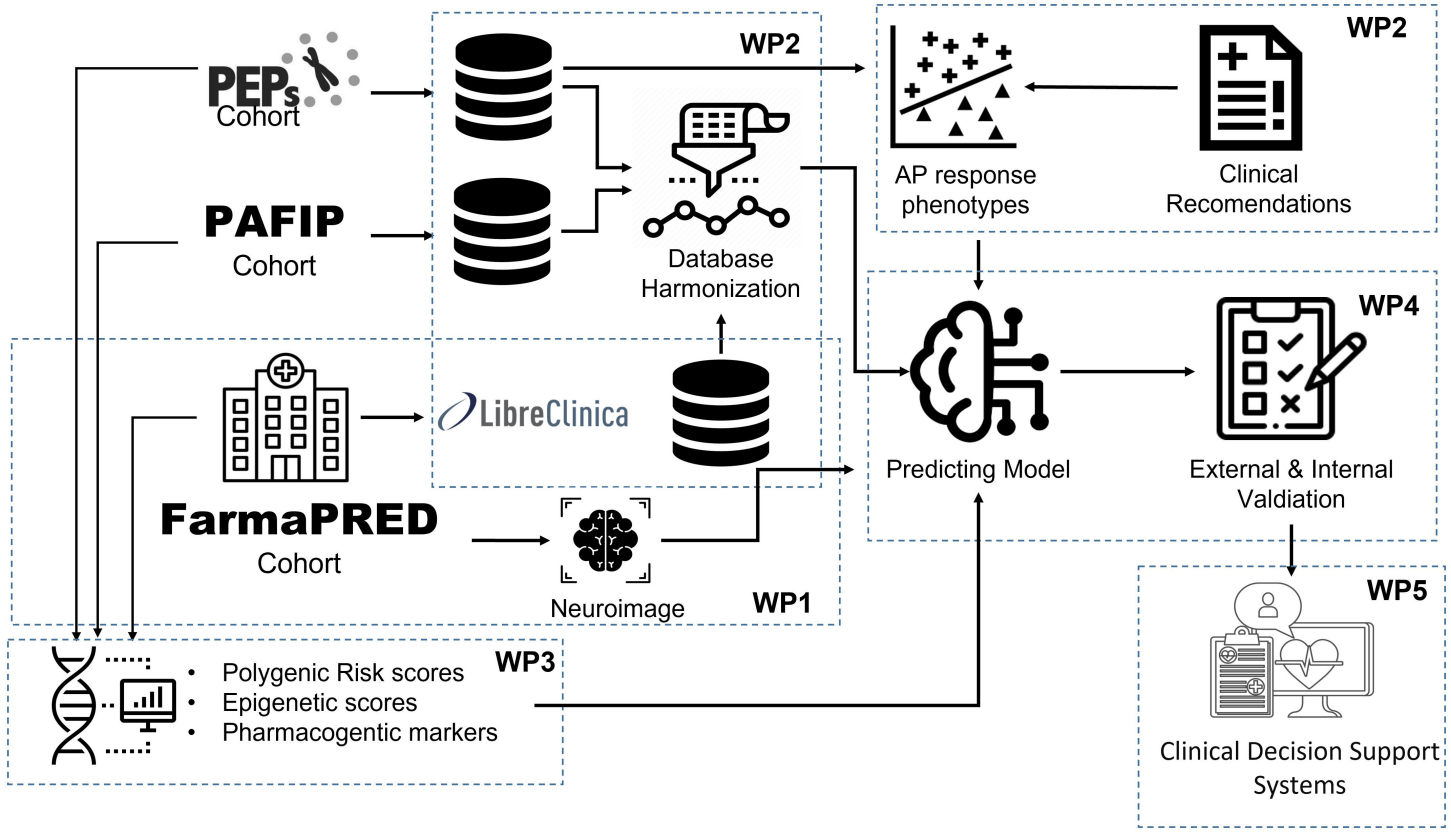
FarmaPRED-PEP study design and workflow.

FarmaPRED-PEP is an observational, naturalistic, and longitudinal study examining clinical trajectories and the predictors of clinical response to APs in FEP cohorts (**Figure 1**).

### Project aims

The specific aims of the FarmaPRED-PEP study are to:

1. Define treatment response phenotypes to antipsychotics in two cohorts of patients with first-episode psychosis (N = 700) using statistical techniques applied to longitudinal data on symptomatology, neurocognition, and adverse effects, before validating these phenotypes in a new prospective cohort (N = 300).
2. Reach consensus on clinical recommendations for the defined treatment response phenotypes.
3. Develop and perform internal, external, and prospective validation of the predictive algorithms for the defined response phenotypes using machine learning techniques that integrate pharmacogenetic variables (measured as PRS) and epigenetic data along with clinical, sociodemographic, environmental, and neuroanatomical data.
4. Develop predictive algorithms for antipsychotic responses specifically adapted for each gender.
5. Develop a computer application that contains the predictive algorithms for the treatment response phenotypes and the clinical recommendations for each.
6. Study the feasibility of the clinical applicability of the predictive algorithms in coordination with healthcare systems.
7. Promote educational programs on personalized and precision medicine in psychiatry.
8. Explore strategies to promote access to genomic and health data, and their potential risks and benefits in psychiatric patients.

**Figure 2:**
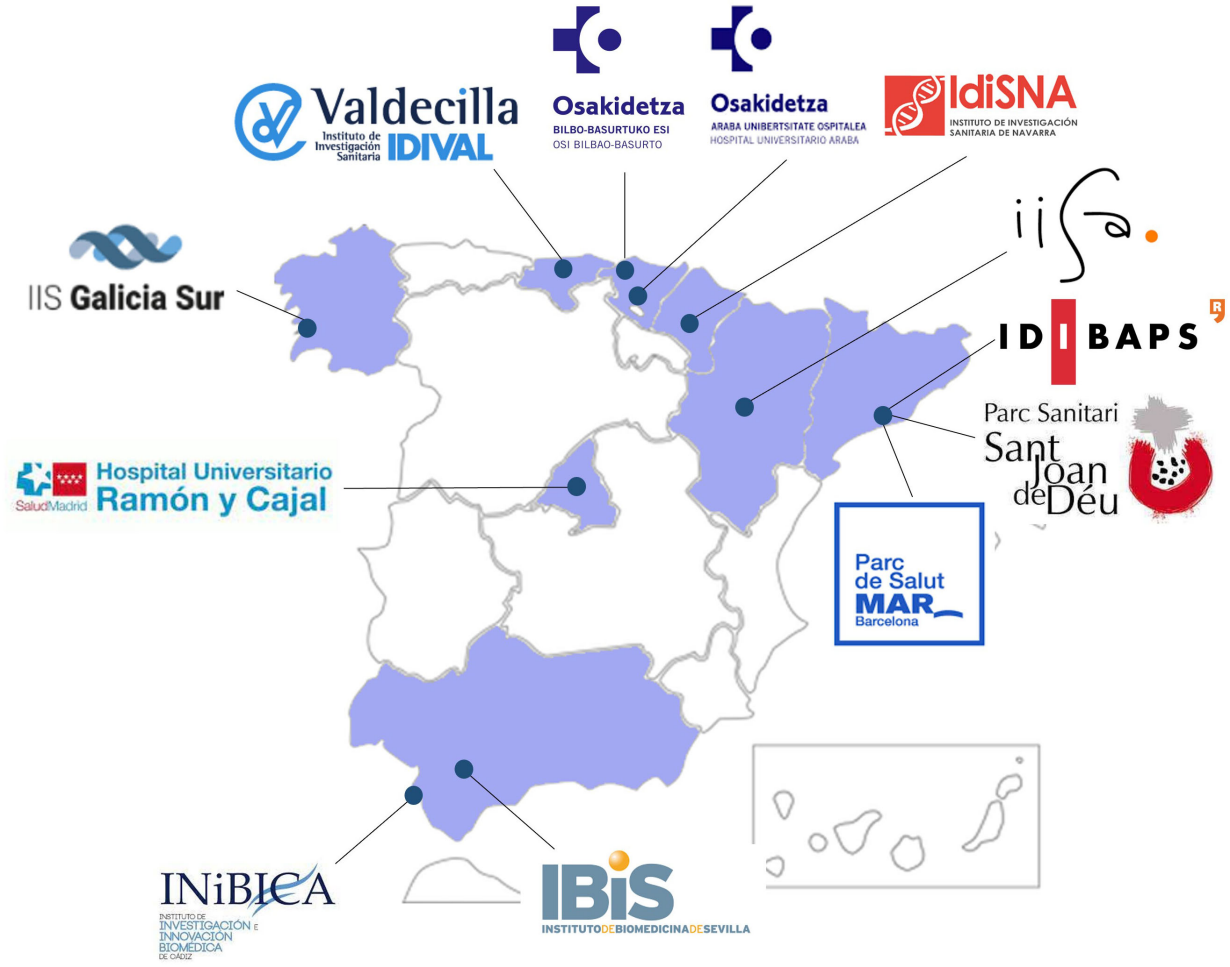
FarmaPRED-PEP research network and study sites.

To achieve these objectives, the FarmaPRED-PEP study has established a national research network that is focused on recruiting FEP patients from 12 national study sites (**Figure 2**), with eight work packages having already been designed (**Table 1**). The teams from these sites are members of the Biomedical Research Networking Center in Mental Health (CIBERSAM from the Spanish abbreviation), a Spanish network focusing on translational research on the neuroscientific aspects related to health and mental illness (www.cibersam.es) (32).

**Table 1:** FarmaPRED-PEP work packages.

Work package	Function
**WP1. FarmaPRED cohort recruitment**	**Task 1.** Decide clinical assessments and time-points **Task 2.** Design and develop a web-based EDC **Task 3.** Recruit 300 patients experiencing FEP at 12 clinical centers. Longitudinal follow-up will consist of four visits (baseline, at 3, 6 and 12 months after study inclusion) during which clinical and neuropsychological assessments will be conducted and the occurrence of adverse effects and plasma levels of APs will be assessed. At the baseline visit, sociodemographic data and family history will be collected, a biological sample for DNA extraction will be obtained, and magnetic resonance imaging (MRI) will be performed.
**WP2. Definition of response phenotypes and clinical recommendations**	**Task 1.** Develop data preprocessing protocols, including data transformation, data imputation, and data harmonization, among the three cohorts included in the present study. **Task 2.** Define response phenotypes to APs in FEP using 12-month longitudinal data on symptomatology, neurocognition, and adverse effects through clustering and latent variable analyses. **Task 3.** Develop a guide of clinical recommendations for each of the response phenotypes.
**WP3. Pharmacogenetic study**	**Task 1.** Develop and apply quality control protocols for genetic and epigenetic data. **Task 2.** Calculate selected polygenic risk scores using whole-genome genotyping following previously described standard protocols. **Task 3.** Calculate epigenetic clocks and epigenetic risk scores using whole-genome methylation data according to standard protocols. **Task 4**. Perform a pharmacokinetic study of the candidate genes involved in the absorption, distribution, metabolism, and excretion of APs and its relationship to AP plasma levels.
**WP4. Development and validation of predictive algorithms**	Develop predictive algorithms of the identified AP response phenotypes using baseline data. The PEPS cohort will be used to create the prediction model and the PAFIP cohort will be used as an independent validation sample. The most robust model will be selected and fine-tuned by merging the PEPS and PAFIP cohorts before being externally validated using the FarmaPRED cohort. Explainable artificial intelligence (AI) methods that derive informative predictions will be used.
**WP5. Development of a clinical decision support system**	In coordination with a specialized company, a computer application that integrates the predictive algorithms (WP4) and the clinical recommendations (WP2) for the identified response phenotypes will be developed. This application must integrate the various algorithms and recommendations sequentially: (1) prediction of symptom trajectory; (2) prediction of neurocognitive trajectory; (3) prediction of adverse effects; (4) therapeutic strategy recommendations and APs; and (5) dosing recommendations and the need for monitoring plasma levels.
**WP6. Study of clinical applicability and feasibility of integration into the Spanish National Health System**	In coordination with the territorial mental health managers of each participating autonomous community, both the clinical applicability and integration into the Spanish National Health System of the findings from this study will be assessed.
**WP7. Education and teaching of personalized and precision medicine in psychiatry**	A program will be developed to promote the teaching of personalized and precision medicine in psychiatry using the network of CIBERSAM centers as a promotion platform.
**WP8. Dissemination of results**	Preparation of publications and communications.

### Recruited samples

The FarmaPRED-PEP study will take advance of two already recruited Spanish cohorts of FEP patients with longitudinal assessment and is currently recruiting a third cohort. The two cohorts previously recruited are: the PEPS cohort (N = 335) (30) and the PAFIP cohort (N = 350) (31). A complete description of these cohorts can be found elsewhere (30, 33).

### Prospective sample

The FarmaPRED-PEP research network is recruiting the FarmaPRED cohort. The inclusion criteria are: aged between 16 and 35 years at the time of first evaluation; a duration of positive symptomatology that does not exceed 12 months; a duration of AP treatment that does not exceed 3 months; fluency in Spanish; and signed informed consent for the study (for minors, by a legal guardian if the patient agrees to participate). The exclusion criteria are: a neurological disorder; traumatic brain injury with a loss of consciousness; intellectual disability, not only an IQ < 70, but also poor functioning; somatic pathology with a mental impact; toxic psychosis; or a refusal to undergo genetic testing.

The study was approved by the research ethics committees of all the participating clinical centers (HCB/2022/0079). Informed consent is to be obtained from all the participants. Informed consent has been designed according to the recommendations of the Spanish Precision Medicine Infrastructure Associated with Science and Technology (IMPaCT) (https://impact.isciii.es/) and the Carlos III Health Institute (Spain) to ensure proper data sharing and to promote open science.

### Primary clinical endpoint

Clinical trajectories, derived from longitudinal data that characterize AP response phenotypes, will serve as the primary clinical endpoint. These trajectories will utilize data collected at baseline, 3, 6, and 12 months and will be identified via clustering and latent variable analyses. Response phenotypes will be based on longitudinal changes across multiple domains, including psychotic symptomatology (positive, negative, and general symptoms), affective symptoms, and side effects. All measures used to define these trajectories are detailed below.

## Methods

### Diagnosis

The diagnosis of a psychotic disorder will be established using semi-structured interviews based on the age of the patient: the Schedule for Affective Disorders and Schizophrenia for School-Age Children - Present and Lifetime Version (K-SADS-PL) (< 18 years old) (34) and the Mini-International Neuropsychiatric Interview (MINI) (> 18 years old) (35). To retrospectively characterize and date the initial symptoms of a psychotic illness, the Symptom Onset in Schizophrenia (SOS) inventory will be used (36).

### Demographic and environmental factors

At baseline, a complete personal and family history will be taken, including a history of drug use. Environmental factors and stressors will also be recorded using several measures that include the Lewis-Murray Obstetric Complications Scale (37), the List of Threatening Experiences Questionnaire (38) and the Childhood Trauma Questionnaire (39), with information on urbanicity and the socioeconomic status also being collected using the Hollingshead-Redlich index. The Premorbid Adjustment Scale (PAS) (40) will be used to assess premorbid adjustment, one of the most studied factors in relation to the prognosis of psychotic disorders. In each evaluation, weight, height, the body mass index (BMI), blood pressure, and the abdominal perimeter will also be recorded, with the aim of monitoring the physical health indicators.

### Treatment data

In each evaluation, information on the drugs prescribed will be recorded, including the type of drug, the duration of treatment, and dosage. Information on psychological treatment will also be collected. To assess adverse drug reactions, several measures will be included: the Udvalg für Kliniske Undersogelser (UKU) side effect rating scale (41), the Simpson-Angus scale, and general blood tests (total cholesterol, LDL, HDL, triglycerides, glucose, and prolactin). AP plasma levels will be measured at each visit. The analysis of plasma levels together with the complete genotyping of the cytochromes and transporters will allow the identification of genuine non-responders, differentiating them from non-adherers to treatment. Adherence will be measured using the Morisky Green Levine Medication Adherence Scale (42).

### Clinical measures

At baseline and in each evaluation, a comprehensive assessment of psychopathology will be performed that will include well-established measures such as the Positive and Negative Syndrome Scale (PANSS) (43), the Brief Negative Symptom Scale (BNSS) (44), the Young Mania Rating Scale (YMRS) (45), and the Montgomery—Asberg Depression Rating Scale (MADRS) (46). The assessment of global functioning will include the Clinical Global Impression (CGI) Scale (47), the Global Assessment of Functioning (GAF) Scale and Functional Assessment Staging (FAST) (48).

### Cognition

The neuropsychological assessment battery will be applied in the three-month evaluation to ensure that the patient is clinically stable. The neuropsychological assessment battery will be repeated in the one-year follow-up visit. The study will use the MATRICS Consensus Cognitive Battery (MCCB) (49) consisting of 10 individually administered tests that measure cognitive performance in seven domains: speed of processing (BACS: symbol coding; category fluency: animal naming; Trail Making Test - Part A), attention/vigilance (CPT-IP), working memory (WMS^®^-III: Spatial Span; Letter-Number Span), verbal learning (HVLT-R™), visual learning (BVMT-R™), reasoning and problem solving (NAB^®^: Mazes), and social cognition (MSCEIT™: Managing Emotions). The MCCB will be complemented by several tests to facilitate the harmonization of the cognitive data among the cohorts. These tests include: the Vocabulary and Matrix Reasoning subtests of the Wechsler Adult Intelligence Scale – Fourth Edition (WAIS-IV) (50) to measure current IQ; the Backward Digit Span subtest of the WAIS-IV (50) to measure working memory; and the Trail Making Test - Part B (51) to measure executive functions. Additionally, at baseline, the Cognitive Reserve Assessment Scale in Health (CRASH) (52) will be used to measure cognitive reserve.

### Neuroimaging

The multimodal neuroimaging protocol is designed for a 3T MRI scanner and includes structural imaging and resting-state functional MRI (rfMRI). Images will be collected at baseline or in the three-month evaluation to ensure that the patient is clinically stable. There are currently 11 MRI scanning sites with a variety of vendors and platforms. Data will be collected from each center and processed at one site. Upon arrival at the data analysis center, quality control (QC) will check the consistency of the data and adherence to the specified imaging protocol. Following QC, the data will be mathematically harmonized to eliminate residual site effects and will then be processed using robust analysis streams.

### Genetics and epigenetics

In all evaluations, blood samples will be collected in whole-blood EDTA tubes. The blood samples will be processed using a standardized protocol to obtain aliquots of plasma (for plasma AP assessment) and buffy coat (for DNA extraction). Samples will be stored in -80°C freezers. The relevant metadata will include the fasting duration, the time since the last AP dosage, and recent illnesses or inflammatory conditions. DNA will be extracted from buffy coat samples following established standard protocols. Normalized DNA concentrations will be sent to the Spanish National Genotyping Center (CeGen). Whole-genome genotyping will be undertaken using the Axiom™ Spain Biobank Array (developed in the University of Santiago de Compostela, Spain) (Thermo Fisher Scientific) and data will be analyzed to obtain polygenic risk scores (PRS) for schizophrenia and other psychopathologies as well as other conditions including cognition, personality traits, and metabolic or inflammatory traits. GWAS summary results will be downloaded from the Psychiatric Genomics Consortium and the Science Genetic Association Consortium. Standard pipeline analysis (previously described (25)) will be performed, allowing efficient quality control, relatedness testing, principal component analysis, and genotype imputation. The selected PRSs will be computed using the PRS continuous shrinkage (PRS-CS) software, a Bayesian-based method that infers an updated posterior SNP effect size by applying continuous shrinkage to the discovery GWAS summary statistics. The external linkage disequilibrium (LD) reference panel will be constructed using a publicly available subsample of the UK Biobank. The derived PRSs will be standardized to z-scores with mean 0 and a standard deviation of 1. The genotyping of specific pharmacogenetic genes (including cytochromes and transporters) will be conducted with the PharmacoScan array (Thermo Fisher Scientific). Only genes with meaningful impacts on AP metabolism, as per CPIC levels A–B for gene-drug interactions (https://cpicpgx.org/genes-drugs/), will be included. Genetic variants will be annotated using star (*) alleles, where applicable, via the PharmVar database (https://www.pharmvar.org/). For statistical analysis, genotype-to-phenotype translations will follow consensus recommendations (e.g., CYP2D6) (53) or, if absent, CPIC-defined phenotypes. DNA methylation will be assessed using the Illumina Infinium MethylationEPIC v2.0 kit (Illumina) and analyzed to calculate different epigenetic clocks (54) and methylation profile scores (MPS) (55) according to standard protocols that include quality control, beta-value computation, normalization, and singular value decomposition to identify and remove unwanted sources of variation (56, 57). We will use publicly available data from methylation-wide association studies as a discovery sample to calculate methylation profile scores using similar approaches to those used for the construction of PRS. We developed a code for computing MPS accessible via the following public repository on GitHub: https://github.com/agonse/methylscore.

### Data collection

Phenotypic data will be collected and managed using LibreClinica, an open-source electronic data capture (EDC) system for clinical trials based on the OpenClinica(R) (OC) 3 community edition. A web-based EDC has been designed specifically for the collection of data from all the measures and questionnaires. Real-time data validation and quality rules will be defined to ensure that the data are entered accurately and as completely as possible. Clinical raters will directly enter the questionnaire data using the corresponding data entry forms. Each data entry will then be carefully double-checked by an external project management team that will resolve any discrepancy or query. Standard operating procedures will be established to ensure proper data collection and comparability across the study sites.

### Data analysis

The analyses will focus on two main goals: (1) the characterization of AP response phenotypes using longitudinal data and (2) the prediction of these phenotypes. Clinical trajectories utilizing data from baseline, 3-, 6-, and 12-month follow-ups will identify AP response phenotypes based on the longitudinal change in different psychotic symptom domains (positive, negative, and general) as well as in affective symptoms and side effects. Trajectory analyses will include all the information available from all the time-points and will apply state-of-the-art clustering and latent variable analyses. The PEPS cohort (N = 300) will be used to identify these AP response phenotypes and to perform their clinical characterization. Predictions will be based on the data collected at baseline and the cognition information collected in the 3-month follow-up. To ensure our ability to test the robustness of the prediction models in an unbiased manner, the PEPS cohort will be used to create the prediction model, while the PAFIP cohort (N = 350) will be used as an unseen independent validation sample. Explainable machine learning methods will be used. The most robust model will then be selected and fine-tuned by merging the PEPS and PAFIP cohorts before being externally validated using the FarmaPRED cohort.

### Sample size and power

We used a recently-described method to determine sample size for developing a clinical prediction model using a traditional likelihood-based approach (e.g., logistic regression) (58). Sample sizes were derived for the scenarios of low, medium or high prediction performance (Nagelkerke R-squared value of 0.25/0.40/0.55), with the number of parameters in the model being 10. The minimum expected frequency of the AP response cluster was 15% according to previous studies with the PEPS cohort (59). Using the R package ‘pmsampsize’, we estimated the sample sizes required for binary outcomes. For a binary outcome, there are three criteria for determining sample size: (1) a small overfitting defined by an expected shrinkage of predictor effects by 10% or less; (2) a small absolute difference of 0.05 in the model’s apparent and adjusted Nagelkerke R-squared value; and (3) a precise estimation (within +/- 0.05) of the average outcome risk in the population for a key time-point of interest for prediction. Each criterion may require a different sample size and the chosen sample size is the largest of the three. For this study, when the outcome is considered binary, the criterion with the largest required sample size will be criterion 1 for a low predictive performance and 2 for medium and high performances. Assuming a phenotype frequency of 15% and a maximum of 10 parameters to be included in the predictive model, the minimum sample size for a model is estimated to be between 290 and 310.

## Discussion

The FarmaPRED-PEP project has been designed to overcome several of the limitations identified in PGx studies in psychiatry, including:

1. Sample size. As commented previously, the recruitment of large cohorts with longitudinal follow-ups and deep phenotyping is challenging and difficult to achieve. Here, the aim of the study is to collect data from 1000 patients through the integration of three cohorts of FEP patients: the PEPS cohort, the PAFIP cohort, and the FarmaPRED cohort. The assessment instruments and measurement time-points to be used in the FarmaPRED cohort were decided via a working group discussion to facilitate the harmonization of clinical and neurocognitive data among the cohorts.
2. Phenotype heterogeneity and definition. One factor hindering the identification of predictors for AP response is the inconsistency in how AP response phenotypes are defined across various studies (28, 59). Many researchers adopt binary classifications to measure effectiveness (categorizing subjects as either responders or non-responders) or evaluate toxicity (determining whether an adverse effect is present or absent), although there is no consensus regarding the ideal cut-off values for these variables. Such binary classifications fail to capture the complex nature of AP responses, which are influenced by both the effectiveness of the treatment (encompassing multiple aspects such as clinical symptoms, neurocognitive performance, and quality of life) and the side effects involved. Additional challenges in predicting AP responses include the diverse progression of the disease itself. Predicting response outcomes using patients with chronic conditions introduces more variability and limits the applicability of the findings due to differences in illness duration, diagnostic criteria, and previous treatments. Conversely, patients experiencing their first episode of schizophrenia generally show less variation in their prior use of antipsychotics, making them more appropriate subjects for studying predictors of treatment outcomes (60, 61). The aim of the FarmaPRED-PEP study is to define the phenotype of response to APs using longitudinal data from over the course of one year after the FEP and considering that this response is a complex phenotype encompassing not only the remission of the various symptoms that characterize the pathology, but also the neurocognitive aspects and the emergence of adverse effects.
3. The complexity of the phenotype. The AP response is a complex phenotype with a polygenic basis that could be partially captured using PRS. However, as is the case with other risk factors used in healthcare (e.g., cholesterol levels), PRS have a predictive capacity with limited accuracy, meaning they cannot predict a clinical variable of interest with sufficient precision at the individual level. Even if the PRS captured all the genetic variability in a pharmacological response attributable to common genetic variants, their predictive capacity would still be imperfect, mainly for two reasons. Firstly, genetic factors are not the only risk factors that explain variability in a pharmacological response as there are many other sociodemographic, environmental, clinical, and pharmacological factors involved in this complex phenotype (62, 63). Secondly, PRS only account for the contribution of common genetic variants, each with a small effect, without considering the effects of rare genetic variants, which are less frequent but have a larger impact, or the effects attributable to epigenetic modifications. Although the inclusion of rare genetic variants, identifiable through massive sequencing techniques, is not expected to increase the predictive capacity of the PRS in the short term (13), the inclusion of epigenetics in pharmacogenomic studies is expected to help explain much of the missing heritability, that is, the heritability of a phenotype that is not captured by genetic variants (3). In this regard, variables such as epigenetic clocks have recently been shown to be related not only to schizophrenia, but also to the response to APs (56, 64, 65).
4. The gender perspective. The study assumes a gender-based approach as a cross-cutting axis to improve health interventions. Therefore, its objective is to develop predictive algorithms for the response to AP, especially tailored to each gender. The differences between women and men in the incidence of FEP are well known, but less studied in relation to the response to AP. In this sense, the study will not be limited to considering gender as a simple categorical variable for introduction into statistical models, but aims to conduct an in-depth study of the impact that gender has on pharmacological response in FEP and its interaction with other health determinants (e.g., age, socioeconomic status, educational level, etc.). Therefore, this study will promote the search, detection, and analysis of differences, as well as similarities, between men and women, both in terms of efficacy and in terms of the frequency and type of adverse effects. Thus, it aims to determine whether the improvement in psychotic symptomatology, but also in neurocognitive characteristics, as well as the frequency and severity of adverse effects, are exclusive to one of the two sexes, more prevalent in one of the two sexes, with different characteristics between both sexes, or even if they receive different responses from the system depending on whether they are men or women. To avoid some of the gender biases and common malpractices in health research, the study data will be collected and analyzed disaggregated by gender and will be presented in reports and publications derived in the same way.

Overcoming these limitations, the aim of the FarmaPRED-PEP study is to develop algorithms to predict AP response phenotypes using genetic and epigenetic data (measured as risk scores) together with clinical, sociodemographic, environmental, and neuroanatomical data.

The future of medicine is focused on offering a comprehensive approach to health, taking into account all the determining factors of health or illness. The generation of large amounts of health data has exceeded the capacity to manage all available information in real time; therefore, artificial intelligence can become a support tool for healthcare professionals, who, however, must always have the final say in decision-making. Machine learning systems, and more specifically deep learning ones, are capable of extracting patterns and generating conclusions from a large amount of data using complex formulas and associations, making them less intuitive. This fact limits the understanding and comprehension of the systems by healthcare professionals, making it difficult to integrate them into workflows and generating a lack of confidence in the results they can provide. Our project will be focus in explainable artificial intelligence methods that derive informative predictions that can be mapped back to individual features and biomarkers, as opposed to some deep learning and other “black-box” techniques that may not be appropriate for achieving interpretability.

The application of precision medicine in psychiatry has been defined as a multi-stage process (66), where PRS play a significant role in each stage, such as in: (1) predicting the risk of developing the disorder to design preventive strategies; (2) stratifying patients based on their own characteristics, both clinical and genetic, to identify the most effective treatment, treatment-resistant patients, and susceptibility to developing adverse effects; and (3) optimizing dosage regimens to ensure treatment efficacy and tolerability based on drug kinetics and patient genetic characteristics. The FarmaPRED-PEP study focuses on the second and third stages of this strategy.

Although the FarmaPRED study has been designed to address several limitations commonly found in pharmacogenomics (PGx) studies, certain constraints should be considered when interpreting future findings. Firstly, as a naturalistic study, treatment heterogeneity and the non-random selection of specific treatments could act as potential confounders. Secondly, while allowing prior antipsychotic (AP) treatment of less than three months aims to facilitate sample recruitment and ensure adequate statistical power, it may also introduce variability that could confound clinical trajectories. Thirdly, although treatment adherence will be monitored, no interventions to enhance adherence are planned; as a result, adherence variability may further confound the response phenotypes based on clinical trajectories. Finally, despite the implementation of rigorous data harmonization protocols to ensure comparability across the three cohorts involved in the study, uncontrolled differences between cohorts may still influence the findings.

The application of these strategies in personalized and precision medicine is especially relevant in patients with FEP, as recovery after FEP has become the primary goal of any treatment strategy (61), with their progress during the first year of treatment considered crucial for disease prognosis and relapse prevention. Although SZ is a potentially disabling and serious mental illness, an appropriate multidisciplinary approach to FEP can contribute to complete recovery (67). A critical period of 2 to 5 years after FEP has been defined, during which therapeutic interventions may be more successful in preventing relapses and achieving functional recovery in patients (68). Preventing relapses in the early stages of the disease has become a major challenge due to the critical impact that a relapse can have on functional prognosis (69). Non-adherence to APs and a lack of disease knowledge are very common in patients with FEP and are associated with an increased risk of relapse, leading to a worse disease course and functionality (70–72).

Psychotic disorders encompass schizophrenia, schizophreniform disorder, schizoaffective disorder, brief psychotic disorder, and substance-induced psychotic disorder. Additionally, other mental health conditions, such as major depressive disorder, bipolar disorder, obsessive-compulsive disorder (OCD), posttraumatic stress disorder (PTSD), and autism spectrum disorder, may also present with short- to medium-term psychotic symptoms. Given the genetic pleiotropy shared among these conditions -captured through polygenic risk scores- and the widespread use of APs to treat them, the findings of the FarmaPRED-PEP study have the potential to offer transdiagnostic insights that extend beyond first-episode psychosis (FEP) patients.

Pharmacogenomic studies and personalized and precision medicine in the field of psychotic disorders will need to address the challenges that must be faced to ensure their future applicability in clinical practice. These challenges have been defined by the International Consortium for Personalized Medicine (ICPerMed) (https://www.icpermed.eu/index.php) and include, among others, the management of genomic and health data in terms of ensuring confidentiality as well as access to citizens and researchers, the involvement of health authorities in promoting personalized medicine to facilitate its implementation in healthcare systems, the education and involvement of healthcare professionals to ensure knowledge, access, and application of personalized and precision medicine strategies, the multidisciplinary integration of researchers and clinicians to develop personalized medicine strategies, and the collection of data and their use for a more efficient patient-centered healthcare system. These challenges are also the pillars of IMPaCT (https://impact.isciii.es/) of the Carlos III Health Institute (Spain), the founder of the FarmaPRED-PEP study. The aims of our study are aligned with the mission of IMPaCT to facilitate the effective deployment of precision medicine in the Spanish National Health System, ensuring scientific and technical quality, equity, and efficiency in the use of available scientific resources to meet the needs of the citizenry.
